# *Nocardia globerula* NHB-2 nitrilase catalysed biotransformation of 4-cyanopyridine to isonicotinic acid

**DOI:** 10.1186/2191-0855-2-25

**Published:** 2012-04-26

**Authors:** Nitya Nand Sharma, Monica Sharma, Tek Chand Bhalla

**Affiliations:** 1 Department of Biotechnology, Himachal Pradesh University, Summer Hill, Shimla, 171005, India; 2 Present Address: Division of Plant Protection, Central Potato Research Institute, Shimla, 171005, India; 3 Present Address: Department of Biotechnology, Delhi Technological University, Delhi - 110042, India

**Keywords:** 4-Cyanopyridine, Isonicotinic acid, Isobutyronitrile, Bacterial nitrilase, Biotransformation, Substrate inhibition, Fed batch

## Abstract

Isonicotinic acid (INA) is an important pyridine derivative used in the manufacture of isoniazid (antituberculosatic drug) and other pharmaceutically important drugs. Nitrilase catalysed processes for the synthesis of pharmaceutically important acids from their corresponding nitriles are promising alternative over the cumbersome, hazardous, and energy demanding chemical processes. Nitrilase of *Nocardia globerula* NHB-2 (NitNHB2) is expressed in presence of isobutyronitrile in the growth medium (1.0% glucose, 0.5% peptone, 0.3% beef extract, and 0.1 % yeast extract, pH 7.5). NitNHB2 hydrolyses 4-cyanopyridine (4-CP) to INA without accumulation of isonicotinamide, which is common in the reaction catalysed via fungal nitrilases. The NitNHB2 suffers from substrate inhibition effect and hydrolysing activity up to 250 mM 4-CP was recorded. Complete conversion of 200 mM 4-CP to INA was achieved in 40 min using resting cell concentration corresponding to 10 U mL^-1^ nitrilase activity in the reaction. Substrate inhibition effect in the fed batch reaction (200 mM substrate feed/40min) led to formation of only 729 mM INA. In a fed batch reaction (100 mM 4-CP/20min), substrate inhibition effect was encountered after 7^th^ feed and a total of 958 mM INA was produced in 400 min. The fed batch reaction scaled up to 1 L and 100% hydrolysis of 700 mM of 4-CP to INA at 35°C achieved in 140 min. The rate of INA production was 21.1 g h^-1^ mg_DCW_^-1^. This is the fastest biotransformation process ever reported for INA production with time and space productivity of 36 g L^-1^ h^-1^ using a bacterial nitrilase.

## Introduction

Isonicotinic acid (INA) is an important pyridine derivative used in the synthesis of antituberculostatic drug isoniazid (isonicotinic acid hydrazide). The conventional chemical process which utilizes 4-cyanopyridine and hydrazine hydrate as reactants is hazardous, energy demanding and expensive ([Yadav et al. 2005]). Recently ethyl isonicotinate (synthesized from INA) has been used for chemoenzymatic synthesis of isoniazid by lipase-catalyzed transesterification in non-aqueous medium onto hydrazine hydrate ([Yadav et al. 2005]). Isoniazid also inhibits the developmental stages of malarial parasites (*Plasmodium gallinaceum* and *P. berghei*) in mosquito ([Arai et al. 2004]). INA is used for the synthesis of inabenfide, a plant growth regulator. Terefenadine, an antihistamine and nialamide, an antidepressant are also derived from INA ([Scriven et al. 1998]). INA also finds application as an anticorrosion reagent, electroplating additive, and photosensitive resin stabilizer ([Wu et al. 1991]).

INA is manufactured through several chemical methods including potassium permanganate oxidation, air oxidation, and ozone oxidation ([Qiao et al. 2000]). An alternative method is the electrolytic method using 4-methylpyridine as the raw material and aqueous sulphuric acid solution as the supporting electrolytes ([Wang et al. 2006]). In contrast to the chemical routes, the enzymatic processes require mild reaction conditions and are gaining popularity in the chemical industry for the synthesis of commodity/fine chemicals. Ease of biocatalyst production, rate of enzyme activity, substrate, and product tolerance are the main factors considered for the commercialization of the enzyme based bioprocesses. The important requirement of a bioprocess is the starting material (substrate), which is either manufactured chemically or available from biotic sources.

Nitrilase mediated hydrolysis of nitriles has been explored by various workers for the synthesis of many pharmacologically important acids. The efforts of academia and industries made possible the utilization of nitrilases for the manufacture of various chiral acids otherwise difficult to produce through chemical route, viz. 5-hydroxypyrazine-2-carboxylic acid, 6-hydroxypicolinic acid, (*R*)-mandelic acid, (*R*)-3-chloromandelic acid, (*R*)-4-cyano-3- hydroxybutyric acid, and (S)-ibuprofen ([Liese et al. 2000]; [Brady et al. 2004,Breuer et al,. 2004]; [DeSantis et al. 2003,Wang 2005,Sheldon et al. 2007,Xue et al. 2011]).

The potential of fungal nitrilases (*Aspergillus niger* K10 and *Fusarium solani* O1) for the bioconversion of 4-CP to INA have been explored by [Vejvoda et al. (2006]) and [Malandra et al. (2009]). Isonicotinamide formed in the reaction catalyzed by the fungal nitrilase decreased the purity INA in the end, which was further hydrolysed to INA utilizing amidase from *Rhodococcus erythropolis* A4. A cascade of immobilized nitrilase and amidase was created for hydrolyzing 4-CP to INA ([Vejvoda et al. 2006]). [Malandra et al. (2009]) utilized the CLEAs (cross linked enzyme aggregates) of purified fungal nitrilases and bacterial amidase for developing CSMR (constant stirred membrane reactor) cascade for improving the purity of INA up to 99.9%.

The nitrilases from the bacterial origin have been unexplored for hydrolysing 4-CP to INA, whereas its isomer 3-CP has been used as substrate for the synthesis of nicotinic acid ([Mathew et al. 1988,Vaughan et al. 1989, Almatawah and Cowan, 1999,Prasad et al. 2007,Sharma et al. 2006,^2011^]). *N. globerula* NHB-2 harbours three nitrile metabolizing enzymes, viz., nitrilase, nitrile hydratase, and amidase ([Sankhian et al. 2003,Bhalla and Kumar 2005]). Nitrilase of *N. globerula* NHB-2 (NitNHB2) is selectively expressed in growth medium supplemented with isobutyronitrile or propionitrile ([Bhalla and Kumar 2005,Sharma et al. 2006,^2011^]). The efforts for the synthesis of non chiral acids using nitrilases are limited in literature, and have been skewed towards synthesis of chiral acids of pharmaceutical importance in recent years ([Sheldon et al. 2007,Liang et al. 2008,Novill et al. 2011,Xue et al. 2011,Pandey et al. 2011]). Keeping in view that non chiral acid of pharmaceutical value are equally important, the NitNHB2 have been explored for the synthesis of nicotinic acid from 3-CP ([Sharma et al. 2006,^2011^]). The NitNHB2 also showed 87% activity for 4-CP hydrolysis with respect to 3-CP without forming isonicotinamide as intermediate. Thus, the present research work was undertaken to explore the potential of NitNHB2 for the synthesis INA from 4-CP. This is first report of utilizing bacterial nitrilase for the production of INA form 4-CP at a fastest rate.

## Materials and Methods

### Materials

4-CP (99 % pure) and INA was from Alfa Aesar, A *Johnson Matthey Company* (earlier *Lanchaster Synthesis*). The media components were procured from HiMedia, Mumbai (India). All the other reagents were of analytical or HPLC grade as per the requirement.

### Microorganism and culture conditions

*N. globerula* NHB-2 (MTCC 6278) was isolated in the Department of Biotechnology, Himachal Pradesh University, Shimla ([Bhalla and Kumar, 2005]). Nitrilase of *N. globerula* NHB-2 was hyperinduced through multiple feeding of isobutyronitrile in the growth medium containing 1.0% glucose,0.5% peptone, 0.3% beef extract, and 0.1 % yeast extract (pH 7.5) described previously ([Sharma et al. 2011]). After 30 h of incubation the cells were harvested by centrifugation at 8,000 x *g* (4°C, 5 min), and washed twice with 0.1 M NaH_2_PO_4_/Na_2_HPO_4_ buffer (pH 7.5), finally resuspended in the same buffer (referred to as resting cells).

### Nitrilase assay

If not otherwise mentioned, the nitrilase assay was performed in reaction mixture (1.0 mL) containing 0.1 M NaH_2_PO_4_/Na_2_HPO_4_ buffer (pH 7.5), 50 mM 4-CP and resting cells at 35°C in a water bath shaker. After 15 min of incubation, the reaction was quenched with equal volume of 0.1 N HCl.

### Analytical methods

Quantitative estimation of 4-CP and INA in the assay mixture was performed through high pressure chromatography (HPLC) using *series 200 Ic pump* (Perkin Elmer) equipped with Inertsil® ODS-3 column (5μm, 4.6x150 mm, GL Sciences, Japan) and *785A Programmable Absorbance Detector* (Applied Biosystem). Chromatogram was monitored at 230 nm using mobile phase (4:1 = 10 mM KH_2_PO_4_, pH 2.8, adjusted with H_3_PO_3_ : acetonitrile) at a flow rate of 1.0 mL per min using NetWin Software (Netel Chromatographs, India). The calibration curves for 4-CP (2-20 mM) and INA (0.2-2.0 mM) was prepared.

One unit of nitrilase activity was defined as that amount of resting cells (mg dry cell = mg_DCW_) which catalyzed the increase of one micromole of INA per min by the hydrolysis of 4-CP under assay conditions.

### Production of isonicotinic acid from 4-cyanopyridine

The optimization of reaction conditions to produce nicotinic acid using NitNHB2 showed stability of the enzyme at 35°C in 0.1 M NaH_2_PO_4_/Na_2_HPO_4_ buffer (pH 7.5) (Sharma et al. [2011]). The other parameters for INA production were optimized in this study.

### Effect of 4-cyanopyridine on nitrilase activity

Different concentrations of 4-CP ranging from 10 to 250 mM were added in the reaction to study the effect of substrate concentration on enzyme activity. The reaction was performed at 35°C for 15 min. NitNHB2 hydrolysing activity in reaction containing 50 mM 4-CP at 35°C was used to gauge the biocatalyst units (U) to be used in subsequent reactions.

### Time course of 4-cyanopyridine conversion

NitNHB2 activity was inhibited at higher concentrations 4-CP, i.e. substrate inhibition effect was encountered. To achieve 100% hydrolysis of 4-CP, NitNHB2 (2.5, 5.0 and 10 U mL^-1^) was added to the reaction mixture (2.0 mL) containing 200 mM 4-CP. The reaction was performed at 35°C and INA formed due to nitrilase activity was quantified every 10 min interval.

### Fed batch reaction at 40 mL scale (200 mM 4-cyanopyridine feed/40 min)

Using 10 U mL^-1^ nitrilase activity in the reaction, 100% conversion of 200 mM of 4-CP to INA was achieved in 40 min. Thus fed batch reaction performed in 40 mL volume containing 10 U mL^-1^ NitNHB2, and 200 mM 4-CP (1.25 g) fed after every 40 min interval. A total of 10 feeds were added to the reaction, and sample was withdrawn before every feed for 4-CP and INA quantification.

### Fed batch reaction at 40 mL scale (100 mM 4-cyanopyridine feed/20 min)

The feeding of 200 mM 4-CP resulted in retardation of NitNHB2 activity after 3^rd^ feed. Thus, a lower level of substrate feeding (100 mM) was applied at an interval of 20 min to achieve higher yield of INA. In 20 feeds, a total of 8.34 g 4-CP was added to the reaction mixture.

### Fed batch reaction at 1 L scale (100 mM 4-cyanopyridine feed/20 min)

The above experiment resulted in 100% conversion of 4-CP to INA up to seventh feed after which the rate of substrate hydrolysis declined. This reaction was scaled up to 1 L in a 1.5 L BioFlow C-32 fermenter (New Brunswick Scientific, USA). The substrate corresponding to 100 mM (10.41 g) was fed after every 20 min. A total of 0.7 mol (72.9 g) of 4-CP was added to the reaction mixture in seven feeds. The temperature of the reaction was maintained at 35°C and impeller speed was 200 rpm for proper mixing of substrate and resting cells.

## Results

The bacterial nitrilase has been for the first time utilized to develop biotransformation process for the production of INA from 4-CP. NitNHB2 activity profile against various nitrilase has been published in our pervious article ([Sharma et al. 2011]). The hyperinduced resting cells of *N. globerula* NHB-2 showed 5.71 U mg_DCW_^-1^ (87% with respect to 3-CP) nitrilase activity for 50 mM 4-CP in reaction mixture at 35°C. The nitrilase of *R. rhodochrous* J1 exhibited 79% activity (0.72 U mg_DCW_^-1^) for 4-CP in comparison for activity against 3-CP ([Mathew et al. 1988]).

### Production of isonicotinic acid from 4-cyanopyridine

#### Effect of 4-cyanopyridine on nitrilase activity

**Figure 1:**
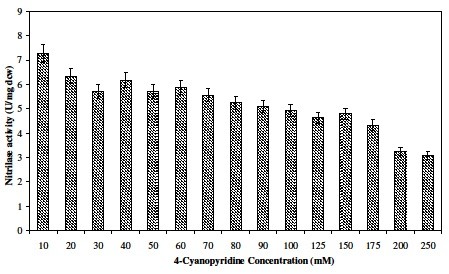
**Effect of 4-CP concentration on nitrilase activity of* N. globerula* NHB-2.**

Similar to the effect of 3-CP concentration on nitrilase activity ([Sharma et al. 2011]), at higher concentrations of 4-CP, inhibitory effect on the nitrilase activity of free cells was observed. Though nitrilase was able to tolerate higher concentrations of substrate there was steady decline of activity (Figure 1). The activity obtained at 50 mM 4-cyanopyridine at 35°C (5.71 U mg_DCW_^-1^) was used to gauge the amount of nitrilase units (U) to be added in the subsequent reaction. [Vejvoda et al. (2006]) and [Malandra et al. (2009]) have used very low concentration of 4-CP, 40 mM and 50 mM for producing INA, respectively.

### Time course of 4-cyanopyridine conversion

**Figure 2:**
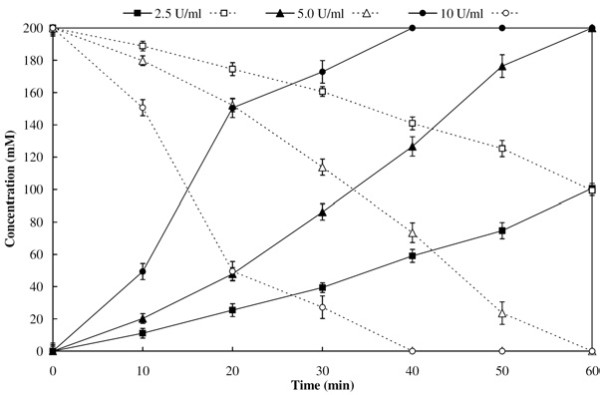
**INA accumulation in the reaction mixture containing 200 mM 4-CP.** The reaction was carried out at 35°C in 2.0 mL reaction containing resting cells corresponding nitrilase units as per the legend (solid legend = INA, hollow legend = 4-CP).

The INA accumulation in the reaction containing 200 mM 4-CP is shown in Figure 2. The added substrate was completely converted to INA, in the presence 5.0 and 10.0 U mL^-1^ nitrilase activity within 60 and 40 min, respectively. These results showed that 200 mM feed at 40 min time interval could be used for fed batch reaction containing 10 U mL^-1^ nitrilase activity.

### Fed batch reaction at 40 mL scale (200 mM 4-cyanopyridine feed/40 min)

**Figure 3:**
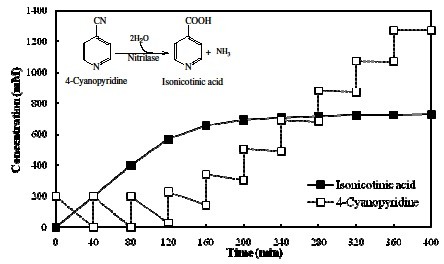
**Accumulation of INA acid and 4-CP in 40 mL reaction mixture containing resting cells corresponding to 10 U mL**^**-1**^** nitrilase activity.** Reaction was carried out at 35°C and 200 mM 4-cyanopyridine was fed at an interval of 40 min.

In presence of 10 U mL^-1^ NitNHB2 in the reaction and feeding 200 mM 4-CP at an interval of 40 min, no substrate inhibition was observed till 2^nd^ feed (Figure 3). The rate of substrate hydrolysis declining to 86% during 3^rd^ feed, producing 172mM INA. In 4^th^ feed the conversion rate further retarded to 43% and only 86 mM 4-CP was hydrolysed. The rate of INA formation in 5^th^, 6^th^, 7^th^, 8^th^ 9^th^ and 10^th^ feed with respect to the 1^st^ feed was 18, 7, 5, 4, 1, and 0%, respectively. A total of 0.729 M INA was formed though a total of 2 M 4-CP was fed. The substrate inhibition effect declined the NitNHB2 hydrolysing activity in this fed batch, leading to the reduction of the biotransformation productivity.

### Fed batch reaction at 40 mL scale (100 mM 4-cyanopyridine feed/20 min)

**Figure 4:**
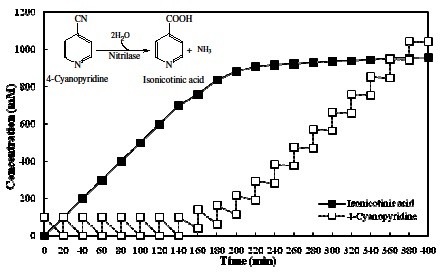
**Accumulation of INA and 4-CP in 40 mL reaction containing resting cells corresponding to 10 U mL**^**-1**^** nitrilase activity.** Reaction was carried out at 35°C and 100 mM 4-cyanopyridine was fed at an interval of 20 min.

The accumulation of INA and 4-CP during 100 mM substrate feeding is shown in Figure 4. The low level substrate feeding resulted in 100% conversion of 700 mM 4-CP to product in 2 h 20 min without affecting the rate of hydrolysis. A similar decline in the rate of 4-CP hydrolysis was observed with respect to previous fed batch, but at higher concentration of INA. In the 8^th^ feed the substrate hydrolysis rate declined to 60%, reaching 10% in the 12^th^ feed and 2% in the last feed. Only 258 mM additional INA was formed though 1300 mM 4-CP had been fed. Low level substrate feeding (100 mM/20 min) improved the productivity and no substrate inhibition was encountered till 7^th^ feed.

### Fed batch reaction at 1 L scale (100 mM 4-cyanopyridine/20 min)

The time and space productivity of any bioprocess is its key feature, ruling the feasibility of its commercialization. The isonicotinic production process was scale up to 1 L (containing 1.75 g_DCW_ corresponding to 10,000 U nitrilase activity), resulting in 100% conversion of the added 0.7 mole 4-CP to INA in 140 min at a rate of 21 g INA h^-1^ g^-1^_DCW_. This biotransformation process is superior to the previously described processes by Vejvoda *et al.* ([2006]) and [Malandra et al. (2009]), which involved use of immobilization of fungal nitrilase and bacterial amidase. *N. globerula* NHB-2 nitrilase achieved the time and space productivity of 36 g L^-1^ h^-1^ INA which is almost 38 times higher than the above processes.

### Recycling of recovered cells

The cells recovered from the 1L fed batch reaction retained 86% of the nitrilase activity (4.92 U mg_DCW_^-1^). These cells when used for a second fed batch reaction (40 mL) successfully converted 700 mM of 4-CP to INA in 2 h 20 min. This showed that the nitrilase of *N. globerula* NHB-2 was stable at 35°C and could be recycled through suitable immobilization techniques. Theoretically it was possible to utilize the recovered nitrilase to produce 0.6 moles of INA (86% of 0.7 moles). Thus, the total theoretical yield of INA through recycling the biocatalyst derived to be 1.3 moles (0.7 + 0.6 mole).

## Discussion

Nitrilase mediated conversion of nitriles to acids are gaining importance over the chemical routes, due to ease of biocatalyst production, mild reaction conditions, and formation of optically pure acids. In past decade, the search of nitrilases for optically active acids have gained importance for both academicians and industries ([Liese et al. 2000,Brady et al. 2004,DeSantis et al. 2003,Breuer et al., 2004,Wang 2005,Sheldon et al. 2007,Xue et al. 2011,Novill et al. 2011,Pandey et al. 2011]). The nitrilases also efficiently hydrolyse non chiral nitriles to acid adding advantage over the chemical routes of their synthesis ([Mathew et al. 1988], Vaughan *et al.* 1989, Almatawah et al. 1999, Vejvoda et al. [2006,Luo et al. 2010,Malandra et al. 2009,Prasad et al. 2007,Raj et al. 2007,Sharma et al. 2006,^2011^]). The current research work was an attempt to explore the potential of *N. globerula* NHB-2 nitrilase for the synthesis of INA from 4-CP for the first time. Further, the study was focused on improving the biotransformation process and scaling up to one liter. [Vejvoda et al. (2006]) have used a cascade of immobilized fungal nitrilase (5.5 U) and bacterial amidase (5 U) on 1 mL Butyl Sepharose column and feeding of 40 mM 4-CP (0.3 mL min^-1^) producing 3.102 g isonicotinic (99.8% purity) in 35 h with time and space productivity of 94 mg L^-1^ h^-1^. In CSMRs cascade loaded with nitrilase (6.5 U) and amidase (5 U) as cell-free extracts immobilized in CLEAs produced 3.36 g INA (99.9%) in 52 h with the time and space productivity of 118 mg L^-1^ h^-1^ when 50 mM 4-CP was pumped at the rate of 10.5 mL h^-1^ (Malandra et al. [2009]). During optimization studies for developing nitrilase mediated biotransformation process using *N. globerula* NHB-2, substrate inhibition effect was encountered, which was partially overcome using low concentration substrate (100 mM) feed. This improved the amount of product formation in comparison to high concentration substrate (200 mM) fed batch. The biotransformation process developed using *N. globerula* NHB-2 nitrilase achieved the time and space productivity of 36 g L^-1^ h^-1^ INA which is almost 38 times higher than above reports. Further, the INA produced was free from isonicotinamide due to lack of hydrating activity of *N. globerula* NHB-2 nitrilase, which was common with the fungal nitrilases.

Since very few INA synthesising biotransformation processes has been documented, comparison with process developed for synthesis of its isomer nicotinic acid, would explain the advantage for current process. The nitrilase mediated processes developed hitherto for the conversion of *N*-substituted aromatic nitriles (cyanopyridines) have not been commercialized due to low substrate tolerance, low product yield and slower conversion rates. The inhibitory effect of 3-CP at 0.3 M and 0.4 M was observed for the nitrilase of *R. rhodochrous* J1 ([Mathew et al. 1988]). The nitrilase of *Nocardia rhodochrous* LL100-21 showed decline in the rate of 3-CP hydrolysis at 0.5 M and above 0.6 M formation of nicotinic acid completely inhibited (Vaughan *et al.* 1989). Further, productivity of nicotinic acid were very low, viz. *R. rhodochrous* J1 (2.29 g nicotinic acid h^-1^ L^-1^ g dcw^-1^), *Rhodococcus* sp. NDB 1165 (8.86 g nicotinic acid h^-1^ L^-1^ g dcw^-1^) ([Mathew et al. 1988,Prasad et al. 2007]). The free cells of *N. rhodochrous* LL100-21 and *B. pallidus* Dac521 immobilized in calcium alginate greatly reduced the nitrilase activity. The nitrilase of these microorganisms remained active for prolonged incubation and only 96 g nicotinic acid in 150 h at a rate of 0.53 g nicotinic acid h^-1^ g_DCW_^-1^ was produced by *N. rhodochrous* LL100-21 (Vaughan et al. 1989). Immobilized free cells of *B. pallidus* Dac521 produced 3.12 g nicotinic acid in 100 h at a rate of 0.104 g nicotinic acid h^-1^ g dcw^-1^ ([Almatawah et al. 1999]).

The present study was an attempt to develop an efficient biotransformation process using *N. globerula* NHB-2 nitrilase for the synthesis of INA. In terms of time and space, the productivity of INA (36 h L^-1^ h^-1^) reported here is the highest. This process is more convenient involving simple fed batch reaction at 35°C in sodium phosphate buffer. The results of the immobilization studies would help to develop a suitable biotransformation process for efficient production of INA.

## Competing interests

The authors declare that they have no competing interests.
